# Carcinoma of the Accessory Axillary Breast: A Diagnostic Dilemma and a Management Challenge

**DOI:** 10.7759/cureus.11844

**Published:** 2020-12-02

**Authors:** Ramanan Sinduja, Ranjith Kumaran, Sudharsanan Sundaramurthi, Balamourougan Krishnaraj, Sarath Chandra Sistla

**Affiliations:** 1 Surgery, Jawaharlal Institute of Postgraduate Medical Education and Research, Puducherry, IND; 2 General Surgery, Jawaharlal Institute of Postgraduate Medical Education and Research, Puducherry, IND

**Keywords:** accessory breast carcinoma, mr mammography, axillary tail, mastectomy, axillary lymph nodes, pathology

## Abstract

Carcinoma of the accessory axillary breast is uncommon, with an incidence of 0.2 to 0.6%. We report a patient whose biopsy of a suspicious lesion in the axilla present for one year revealed invasive ductal carcinoma (IDC). There was presence of breast tissue and absence of lymphoid tissue in the biopsy, suggesting a breast malignancy. Magnetic resonance mammography was suggestive of the lesion well away from the normal breast, confirming an accessory axillary breast. She was offered wide local excision of the lesion with axillary lymph node dissection and modified radical mastectomy (MRM), and she chose the latter. Her post-operative biopsy showed the involvement of all the 25 lymph nodes harvested. Any suspicious lesion in the axilla should be promptly worked up for malignancy. Accessory axillary breast carcinoma, if confirmed, can be addressed with wide local excision along with axillary lymph node dissection. However, further studies should clarify this and the outcomes.

## Introduction

Carcinoma of the accessory axillary breast is uncommon. It could be initially missed if there is no substantial clinical suspicion. Also, if there is skin involvement, there is often less suspicion as the differential diagnoses may be suggestive of a skin pathology such as sebaceous cyst, hidradenitis, lymphadenitis, when in fact, it could represent a higher stage. This delay in diagnosis could have an impact on overall survival. There is a further difficulty in establishing the diagnosis of accessory axillary breast carcinoma, as it needs to be confirmed to decide the management. This is important since non-involvement of the ipsilateral breast or the axillary tail of the ipsilateral breast can be addressed with wide local excision with or without an axillary lymphadenectomy and may not warrant a more morbid and disfiguring modified radical mastectomy. However, future studies need to clarify this and the long-term recurrence and outcome survival rates.

## Case presentation

A 58-year-old post-menopausal lady, a known diabetic and hypertensive, presented with complaints of swelling in the left axilla of a one-year duration. The swelling was insidious in onset, gradually progressive, and was associated with occasional pain. On examination, there was a 3 x 2 cm hard, non-tender swelling in the left axilla. A 1 x 1 cm mobile lymph node was palpable in the left axilla. There was no palpable lump in the left breast. Examination of the right breast and axilla revealed no palpable swellings.

**Figure 1:**
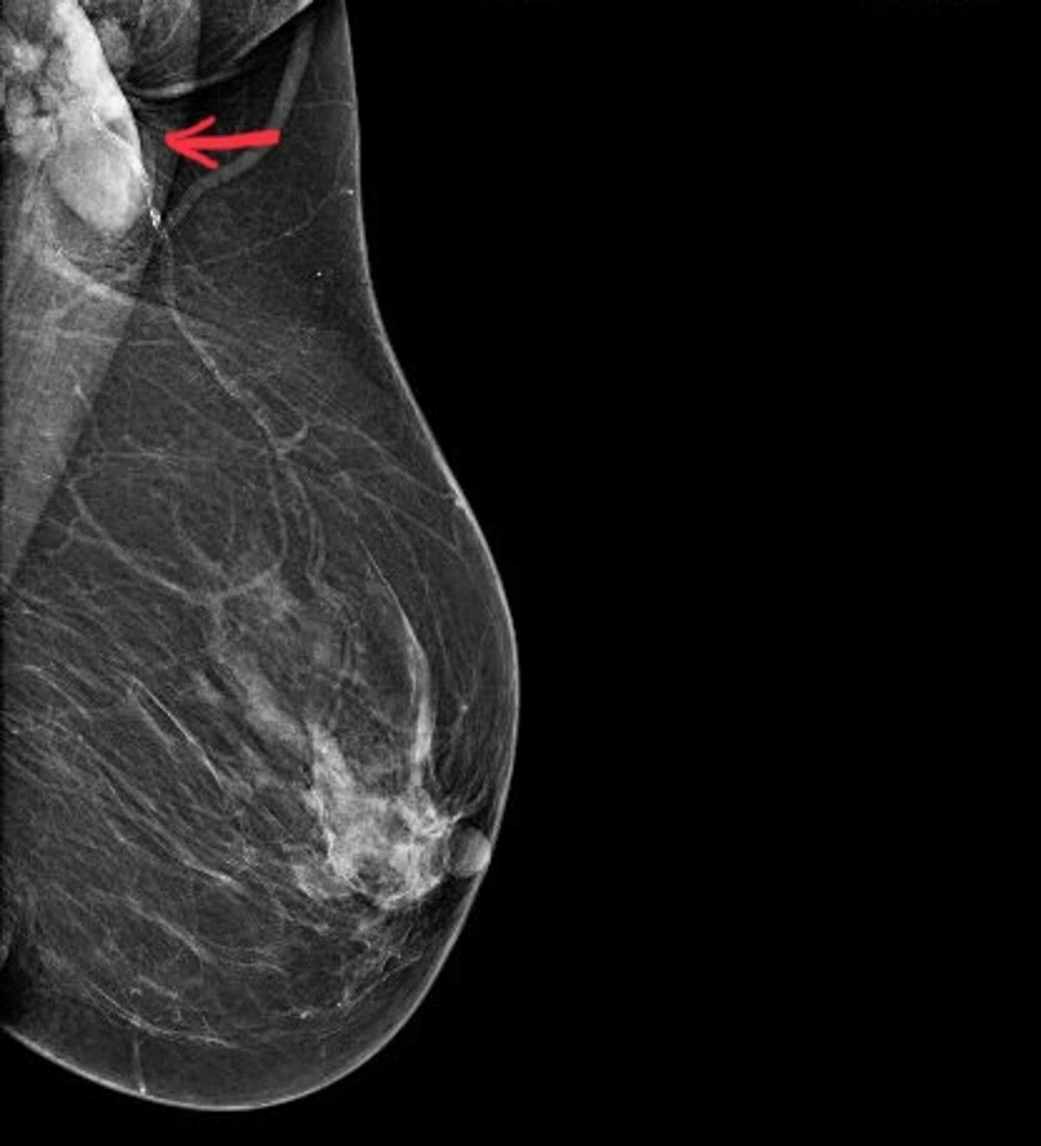
Mediolateral oblique view of left breast and axilla showing an equal to high-density oval lesion in the left axilla. Ultrasonography of the corresponding lesion showed a heterogenous hypoechoic finely spiculated lesion in the left axilla.

Bilateral mammography as an initial modality showed an equal to high-density oval lesion in the left axilla, with few enlarged axillary lymph nodes (Figure 1).

**Figure 2:**
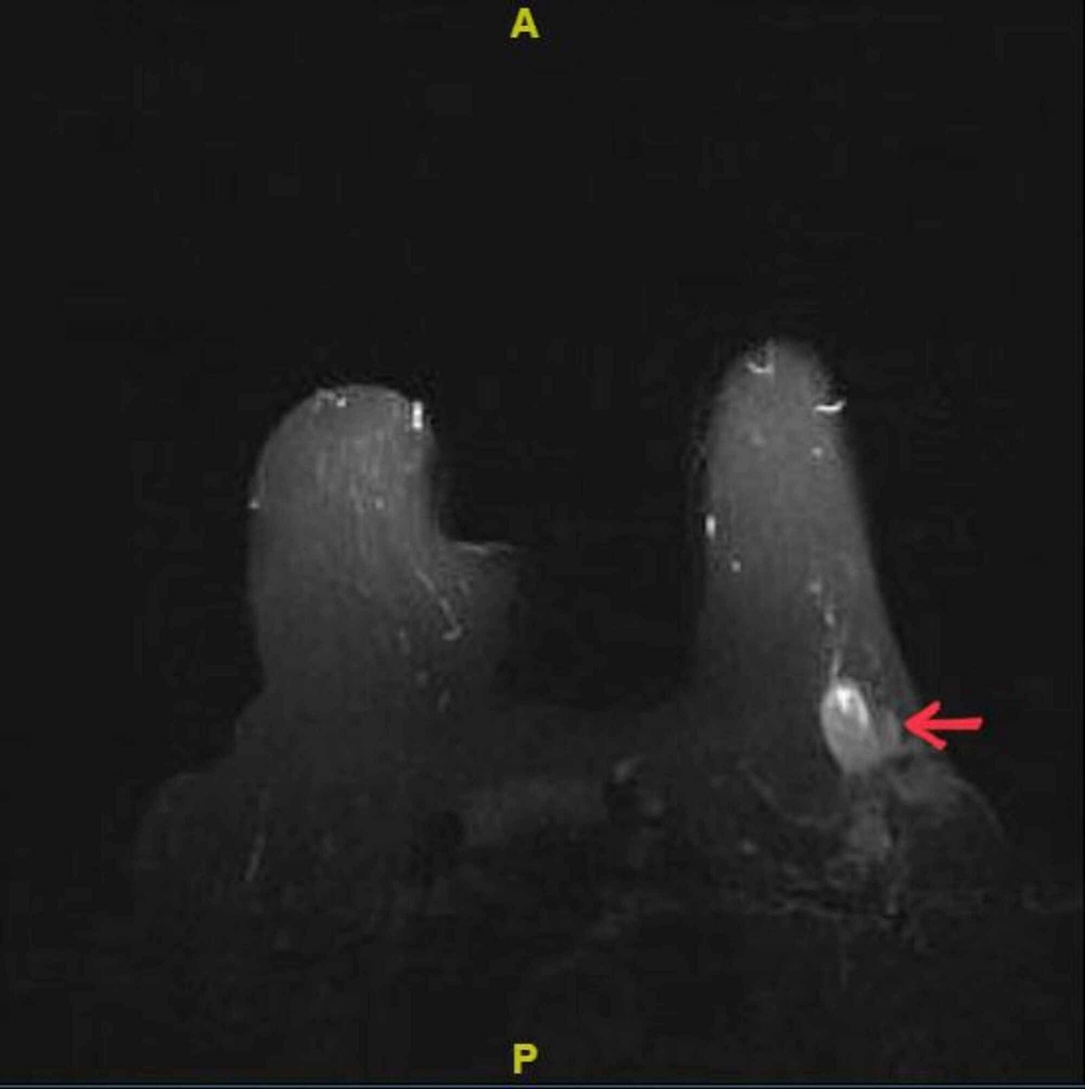
MR mammography showing an irregular well-defined enhancing soft tissue lesion in the left axilla infiltrating the skin. Bilateral breast parenchyma show no abnormal signs or enhancement.

In view of the hard nature of the swelling, core needle biopsy from the swelling was done, which showed the morphology of infiltrating ductal carcinoma (IDC) - not otherwise specified (NOS) of the breast. There was no lymphoid tissue identified. Immunohistochemistry revealed an estrogen receptor (ER) and progesterone receptor (PR) positive, and an HER-2/neu (human epidermal growth factor receptor-2/neu) negative tumor, with a Ki-67 index of 25%. Re-examination of the left breast following the biopsy report did not reveal any lump. In view of the diagnostic dilemma of carcinoma of the axillary tail of the left breast or carcinoma of the left accessory axillary breast, a magnetic resonance (MR) mammography was performed, which revealed a lump in the left axilla with normal left breast (Figure 2).

**Figure 3:**
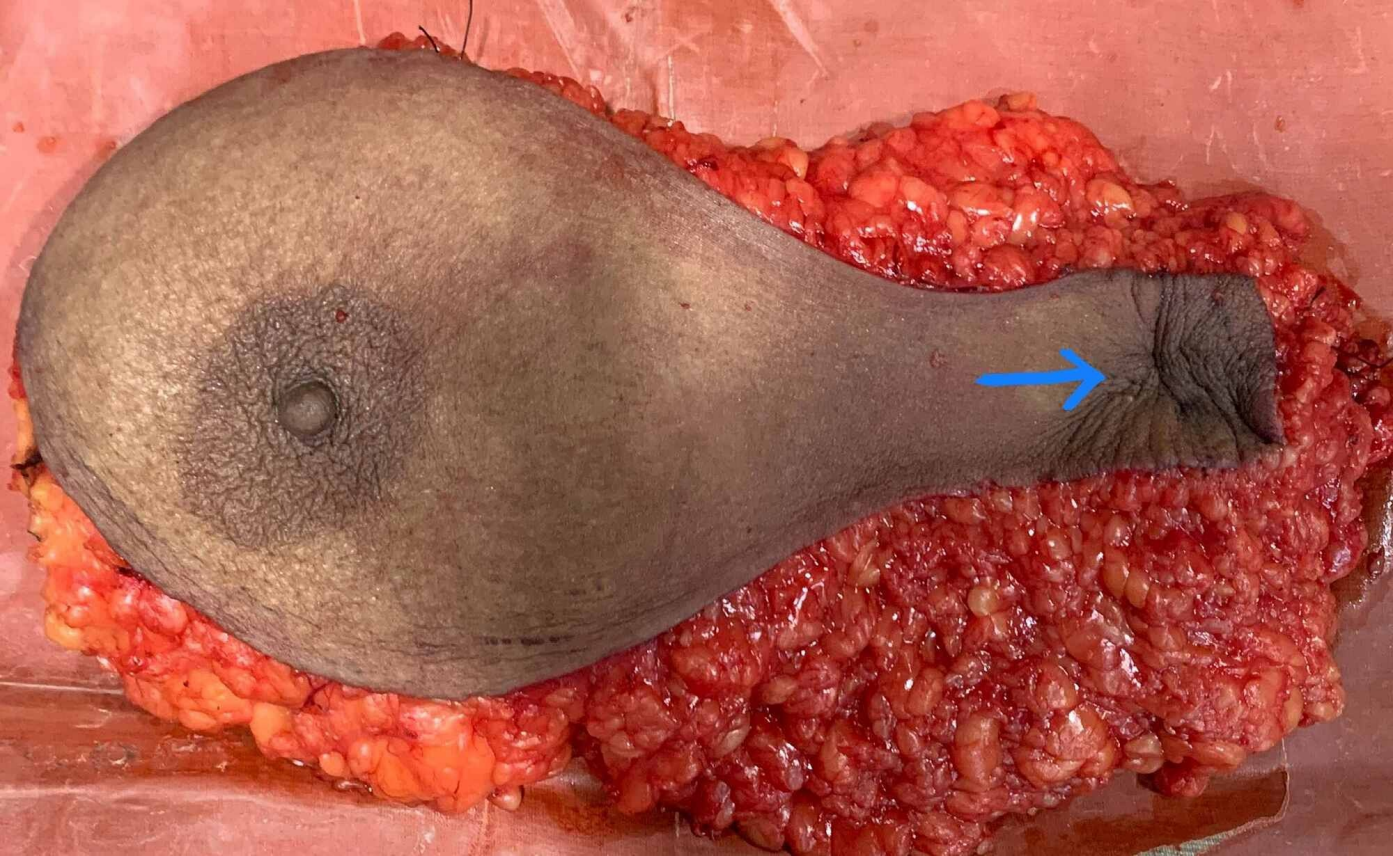
Left mastectomy specimen showing the lesion in the left axilla.

**Figure 4:**
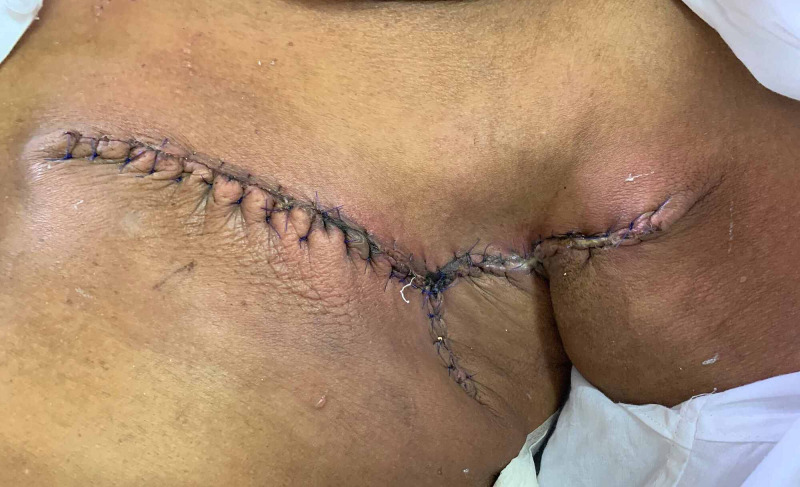
Extended axillary incision used in the surgery.

She was offered a choice of wide local excision of the lesion along with left axillary dissection followed by radiotherapy or a modified radical mastectomy, and the patient opted for the latter. She then underwent left modified radical mastectomy with an extended incision to the left axilla (Figures 3, 4).

The histopathology of the resected specimen showed a 2 x 2 x 1.5 cm tumor in the left axillary pad of fat, with resected margins free of the tumor. Histopathology was suggestive of IDC, and all the 25 axillary lymph nodes harvested were positive for the tumor. Lymphovascular invasion and extranodal extension were present. At one year of follow-up, she had completed her adjuvant chemotherapy - three cycles of FEC (5-Fluoro uracil, Epirubicin, Cyclophosphamide) and four cycles of Docetaxel. She still awaits her radiotherapy due to delay owing to the COVID-19 pandemic. She otherwise has no complaints.

## Discussion

The mammary ridges or the ‘milk line’ run bilaterally on the ventral aspect from the anterior axillary fold down to the groin, ending at the medial aspect of the upper thigh. They involute except to form breasts bilaterally. The persistence of this ectodermal ridge leads to ectopic or accessory breast tissue. However, they may occur outside the milk line as well, in the face, back, or thigh. The prevalence of accessory breast tissue ranges from 2% to 6% in females and from 1% to 3% in males. It is found less in Caucasians than Asians, with the Japanese having a high prevalence of around 5% [1]. They are often noticed when the patient has symptoms, either as swelling or with associated pain.

Kajava in 1915 classified supernumerary breasts into seven different types, containing glandular tissue, nipple, or areola in different combinations. The occurrence of malignancy in this ectopic breast tissue is very uncommon, with a reported incidence ranging from 0.2% to 0.6% [2]. Axilla is the most common site (58%), followed by parasternal (18.5%), subclavicular (8.6%), submammary (8.6%) and vulvar (4%) regions [3]. The suspicion of malignancy is often less in these accessory breasts due to a lack of awareness of the possibility of the accessory breast and its inconsistent appearance. This often leads to a delay in diagnosis, with a higher stage of the tumor at presentation, often with skin involvement [1]. Due to its less incidence, the diagnostic approach and management of accessory breast carcinoma are not well established, with no definitive guidelines.

A mass located in the axilla is the most common presentation in accessory breast carcinoma, with an average diameter of 2.8 cm. Edema, nipple discharge, and even systemic symptoms are noted occasionally. Invasive ductal carcinoma was the most common histopathological type (75%). Nihon-Yanagi et al. showed that the occurrence of mucinous carcinoma (7.8%) and apocrine carcinoma (6.3%) was higher in accessory breast [4]. Marshall et al. further showed a high incidence of infiltrating lobular carcinoma (ILC) (9.5%) in accessory breast [3]. The incidence of these types is high compared to the occurrence in the normal anatomical breast: ILC (3%), mucinous carcinoma (2.9%), apocrine carcinoma (1.1%) [4].

Axillary breast carcinoma occurs more commonly in females, but literature reports an increased incidence of this tumor in males than the usual pectoral location of breast carcinoma [5]. However, the diagnosis of this unusual malignancy can be challenging, keeping in mind a number of differentials that could be a possibility, including axillary lymphadenopathy secondary to benign or malignant etiology, sebaceous cyst, hidradenitis, etc.

Establishing the diagnosis of accessory breast carcinoma could be challenging since its clinical suspicion in an axillary lump is not very high. Initially, an FNAC could help to differentiate between benign and malignant swelling. A core needle biopsy can help to establish the histological type of cancer and the receptor status. Mammography would reveal normal breasts bilaterally, with a suspicious lesion in the axilla. Local ultrasound of the lump could further be done to confirm the axillary location of the lesion.

Once confirmed histopathologically, computed tomography (CT) can be done for metastatic workup, and to know the local extent of the tumor. To further rule out occult breast lesions in both the breasts and any extension of the axillary lesion into the breast, an MR mammogram can be done. This could also help rule out carcinoma of the axillary tail of Spence, which is a part of the upper outer quadrant of the breast, as it may warrant a modified radical mastectomy (MRM). The patient reported in our case report had no evidence of lesion in the bilateral breast in both mammography and MR mammography. Mammography was suggestive of a highly suspicious lesion in the left axilla well away from the normal breast parenchyma on that side. Further, the suspicion of a metastatic lymph node from an occult primary can be ruled out by the presence of breast glandular tissue and the absence of lymphoid tissue in histopathology. We established the final diagnosis of accessory breast carcinoma based on this histopathological principle and the location of the lesion in mammography and MR mammogram. Positron emission tomography (PET)-CT scan can show enhancing areas and can help to confirm the presence of malignancy and the location. However, its role in establishing the diagnosis of accessory breast carcinoma is not yet established, especially when MR mammogram can identify lesions with higher sensitivity [6].

The number of positive axillary lymph nodes remains the strongest predictor of survival in carcinoma of the breast. The rate of nodal positivity in accessory breast carcinoma ranges from 46% to 50%. The concept of early metastasis to adjacent axillary lymph nodes from accessory breast carcinoma, which lie in close proximity, is still not clear [7]. While certain reports advocate this, Nihon-Yanagi et al., in their review of 94 patients in Japan, did not show any higher risk of metastasis to adjacent lymph nodes from this malignancy [5]. However, the post-operative biopsy of the patient reported in this case showed 25 lymph nodes harvested, all involved by the tumor. There was also the presence of lymphovascular invasion and extranodal invasion, suggestive of a very aggressive tumor.

As much as establishing the diagnosis is delayed, deciding on the treatment is also delayed since there are no definitive treatment guidelines. Once the diagnosis of accessory breast carcinoma is established, the stage of the tumor needs to be assessed. Locally advanced tumors can be addressed with neo-adjuvant chemotherapy. Operable tumors are addressed by wide local excision in most reported cases, with or without axillary dissection. In a few cases reported, sentinel lymph node biopsy was done to decide on the axillary dissection [8].

Zhang et al. hypothesized that when the tumor in the accessory breast is located close to the normal breast, indications, and principles of surgery should be the same as lesion located in the normal breast [7]. Literature shows no additional benefit of prophylactic ipsilateral mastectomy and can hence be avoided. Evans and Guyton, in their report where they followed up post-operative patients, showed that in those with no evidence of disease in follow-up, more people had in fact, undergone wide local excision alone [9]. Also, this report showed that early recurrence was comparable in the wide local excision and the radical mastectomy patients, and patients who developed even distant metastasis didn’t have any appearance of the lesion in the ipsilateral breast.

Adjuvant chemotherapy and hormonal therapy are administered on the same principle as the usual pectoral breast carcinoma. Our patient was explained the rarity of this type of carcinoma and the management of the usual breast carcinoma. She was given the choice of wide local excision and modified radical mastectomy, and she chose the latter. Marshall et al. reported a poorer prognosis in accessory breast carcinoma compared to normal breast cancer [3]. They attributed this to a delay in diagnosis and management due to a low clinical suspicion. However, the outcomes of this malignancy are not yet well established due to the rarity of this disease and lack of follow-up.

## Conclusions

Carcinoma of the accessory breast is rare. Further, there is a delay in the diagnosis and management of this malignancy due to low clinical suspicion and a lack of standard guidelines. If the location of the lesion is confirmed to be in the axilla, wide local excision with or without axillary dissection can be done, thereby avoiding ipsilateral mastectomy. Retrospective studies could clarify the different surgical options and their respective outcomes so that patients can confidently choose their options with clarity and without any doubt or anxiety.
